# Microscopic Imaging Methods for Organ-on-a-Chip Platforms

**DOI:** 10.3390/mi13020328

**Published:** 2022-02-19

**Authors:** Bailey C. Buchanan, Jeong-Yeol Yoon

**Affiliations:** Department of Biomedical Engineering, The University of Arizona, Tucson, AZ 85721, USA; baileybuchanan98@email.arizona.edu

**Keywords:** OOC, microfluidic device, transillumination, fluorescence, smartphone-based microscopy

## Abstract

Microscopic imaging is essential and the most popular method for in situ monitoring and evaluating the outcome of various organ-on-a-chip (OOC) platforms, including the number and morphology of mammalian cells, gene expression, protein secretions, etc. This review presents an overview of how various imaging methods can be used to image organ-on-a-chip platforms, including transillumination imaging (including brightfield, phase-contrast, and holographic optofluidic imaging), fluorescence imaging (including confocal fluorescence and light-sheet fluorescence imaging), and smartphone-based imaging (including microscope attachment-based, quantitative phase, and lens-free imaging). While various microscopic imaging methods have been demonstrated for conventional microfluidic devices, a relatively small number of microscopic imaging methods have been demonstrated for OOC platforms. Some methods have rarely been used to image OOCs. Specific requirements for imaging OOCs will be discussed in comparison to the conventional microfluidic devices and future directions will be introduced in this review.

## 1. Introduction

Organ-on-a-chip (OOC) devices mimic human organs, which can be used for many different applications, including drug tests (efficacy and toxicity) [1,2,3,4,5], environmental toxicology [6,7], disease models [8], stem cell differentiation, carcinogenesis [9], etc. [10]. Various analytical methods have been used for OOCs to monitor the number and morphology of cells, gene expression inside the cells, protein secretions outside the cells, and the extent of cell metabolism [10,11]. These methods include various gas and liquid chromatographic analyses, spectroscopic analyses, in vitro immunoassays, on-chip immunosensors, nucleic acid amplification methods (including polymerase chain reaction or PCR), etc. [12]. Microscopic imaging methods are the most popular methods used for OOCs, which can monitor the number and morphology of cells, gene expression, protein secretions, etc. There are a variety of imaging techniques that can be applied to an OOC platform. This review will investigate many examples for three different categories of imaging techniques, including the advantages and disadvantages of each. This review will also discuss the challenges in microscopic imaging from the microfluidic channels within OOCs, e.g., micro-sized samples [13,14]. Microscopic imaging from the conventional microfluidic devices (OOCs have frequently been considered a subset of microfluidic devices) are also included in this review since they are not very different from OOCs. ‘Microfluidic device’ is a more generalized term for devices featuring microfluidic channels which are used for various chemical and biological analytical applications. The applications include point-of-care diagnostics, drug screening, cell analysis, genotyping, etc. [13]. OOCs offer a more specialized function, where the microchannels are seeded with various cells towards mimicking a human organ. Drug toxicity studies are currently one of the primary needs and trends of OOC platforms. OOC platforms offer the advantage of a low-cost option to assess the safety of various drugs and therapeutics [15,16]. A variety of examples, among others, will be provided in this review of how various imaging methods can be combined with OOC platforms to assess parameters such as drug toxicity, cell–cell interactions, and more. The three different categories of imaging techniques for OOCs that will be discussed in this review include transillumination imaging techniques, fluorescence imaging techniques, and smartphone-based imaging techniques, including lens-free smartphone-based imaging.

## 2. Transillumination Imaging

**Figure 1:**
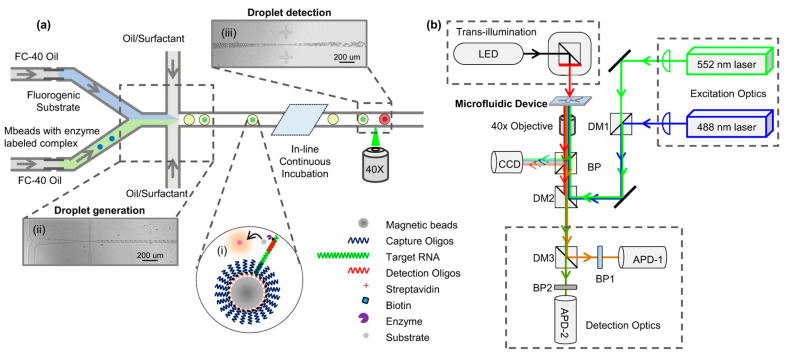
Transillumination imaging of a microfluidic device. Panel (**a**) represents a schematic of the microfluidic device used by Guan et al. Panel (**b**) demonstrates the traditional setup of transillumination imaging where the LED is used as the light source and a microscopic objective lens and a CCD detector on the other side of the microfluidic device that is being imaged. Reproduced from [19] under Creative Commons Attribution 4.0 License.

One of the most standard methods for imaging microfluidic devices and OOCs comes in transillumination. Transillumination is a method that requires a microscope and consists of light being transmitted through a sample on the platform [17,18,19,20,21,22,23]. For transillumination imaging, a light source is needed on one side of the platform, and then a detector is required on the other side [22]. Guan et al. demonstrated a typical transillumination imaging setup with an LED as a light source, the 40× objective as the microscopic lens, and a charged coupled device (CCD) as the detector, as shown in Figure 1b [19]. The light travels through the device and to the other side, hitting the chosen detector to form an image [19]. Sapuppo et al. set up their transillumination imaging around microfluidic devices with a halogen lamp as the light source and a fiber optic as the light collector [24]. Since transillumination does not require labeling the sample—e.g., fixing and staining for fluorescence imaging—cells can be imaged in situ within the microfluidic channels. Such a no-labeling feature is one of the advantages that transillumination offers, which saves time and adds to the ease of the method. Transillumination forms that are commonly used with microfluidic devices include brightfield imaging, phase-contrast imaging, and holographic optofluidic microscopy.

### 2.1. Brightfield Imaging with Microfluidic Devices

Brightfield imaging has extensively been demonstrated for various microfluidic devices. It includes a variety of specialized sub-methods, such as shadow imaging and spatiotemporal image correlation spectroscopy (STICS). Jagannadh et al. utilized brightfield imaging to capture the images of the flow of red blood cells through their glass-based microfluidic device [25]. Instead of traditional polydimethylsiloxane (PDMS)-based microfluidic devices, they demonstrated good accuracy and high throughput imaging even with glass-based microfluidic devices. Lange et al. specifically chose to use shadow imaging by combining a microfluidic chamber with a camera chip and an illumination system to capture shadow images of a nematode [26]. The nematode sample is placed directly on the imaging sensor (camera chip) to produce a shadow image in this method. The image resolution was 320 × 240 pixels. A drawback of this imaging technique is the blur captured in the images; however, Lange et al. found that sufficient lighting could reduce blur. Asghar et al. demonstrated another example of shadow imaging in microfluidic devices [27]. They captured the shadow images of the cells of interest, and automatically processed and counted them using custom software. This is one of the reasons shadow imaging can be beneficial for imaging microfluidic devices as these researchers automatically counted the cells present in the device from the shadow images captured [27]. When thresholded correctly, their software counted the number of cells with higher accuracy compared to manual cell counting, which requires more time to complete [27]. Another type of brightfield imaging is spatiotemporal image correlation spectroscopy (STICS), which combines brightfield imaging with a fast camera [28,29]. Travagliati et al. used this imaging technique to examine the flow velocities of samples in a microfluidic device [29]. They demonstrated a resolution of 5 µm; however, this method could no longer effectively collect flow velocities at low concentrations of particles in the sample. Ceffa et al. used STICS imaging to measure the 3D flow within their microchannels with a micrometer resolution [28]. The downfall was the need for a traditional microscope that is high in cost and not easily transportable. On the other hand, virtual microscopy can offer accessibility of digital images over a large population [30,31]. Transportability becomes essential when developing imaging platforms that can be used in the field.

### 2.2. Brightfield Imaging with OOC Platforms

**Figure 2:**
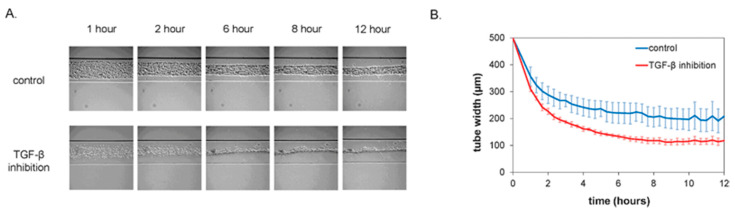
Panel (**A**) represents the brightfield images captured the tubular structures of the pericytes in an OOC platform. Panel (**B**) represents the change in the tube width of the pericytes as a function of time. Reproduced from [32] with permission, © 2013 Royal Society of Chemistry.

Brightfield imaging can also be used specifically to image organ-on-a-chip (OOC) devices. van der Meer et al. created an OOC device with human umbilical vein endothelial cells (HUVECs), human embryonic stem cell (hESC)-derived pericytes, and rat collagen into their microfluidic device to mimic vascular tissue [32]. In this work, the pericytes were labeled to recognize the different cell types once brightfield images were taken [32]. It was found that the brightfield imaging was able to distinguish the tubular structures of pericytes, as seen in Figure 2, but this imaging method did not have good enough resolution for monitoring cell–cell interactions [32]. Another group, Peel et al., modeled how brightfield imaging can be used in OOC platforms to automatically determine the field of view for the second pass of a higher resolution imaging method [33]. This group developed a liver on a chip model where the bottom layer of the microfluidic channel was seeded with endothelial cells and the top layer with hepatocytes. They encompassed all of this in an extracellular matrix [33]. They first obtained the lower resolution brightfield images from the liver on a chip. Following this, higher resolution images were collected to analyze the cellular structures of the liver on a chip [33]. Another group, Agarwal et al., developed a heart on a chip platform for drug testing studies [34]. Agarwal et al. used brightfield imaging to image the microfluidic channels when the heart on a chip was in systole and diastole [34]. These brightfield images were essential to confirm that the heart on a chip was functioning correctly. Overall, brightfield imaging can be beneficial when high-resolution images are not of importance. This method can also provide the first step to ensure the correct structures are formed before putting the time, effort, and resources into obtaining higher resolution images with a different imaging method. However, all of this leads to the major disadvantage of low resolution from brightfield imaging. High resolution is essential when trying to image OOC platforms since the cells and subcellular structures in the device are on a micron to submicron level. Both van der Meer et al. and Peel et al. have found advantages of this imaging method as a preliminary step to imaging OOC devices [30,31], however this imaging technique is not normally the only imaging method used when imaging OOC platforms.

### 2.3. Phase-Contrast Imaging with Microfluidic Devices

Phase-contrast imaging is another method of transillumination imaging that can be used with microfluidic devices [35,36,37]. Phase-contrast imaging is used to observe phase changes in a sample and can be observed through an interferometer or through intensity images [38]. This type of imaging is beneficial for monitoring changes in refractive index as when the sample changes phase it is usually the result of a change in refractive index [38]. Jang et al. developed a system that took advantage of quantitative phase microscopy combined with microfluidic devices. Their system creates a reference field, with the light obtained through the areas of the microfluidic device without channels, and a setup in which a reflection-type spatial light modulator was used to control the phase of these reference fields [39]. This imaging technique could obtain extended field depth, and neither the objective lens nor the sample needed to be scanned [39]. Overall, this imaging method can be beneficial in measuring morphological changes in samples on microfluidic devices [39]. Finally, Kim et al. demonstrated that the combination of a fluorinated polymer to create microfluidic devices with soft lithography can have unique advantages when imaging that device with phase-contrast imaging [35]. This main advantage comes from the similar reflective indexes from the device material and the solution being added to the device [35]. Overall, this imaging method can be beneficial when the phase changes of the sample are of interest.

### 2.4. Phase-Contrast Imaging with OOC Platforms

**Figure 3:**
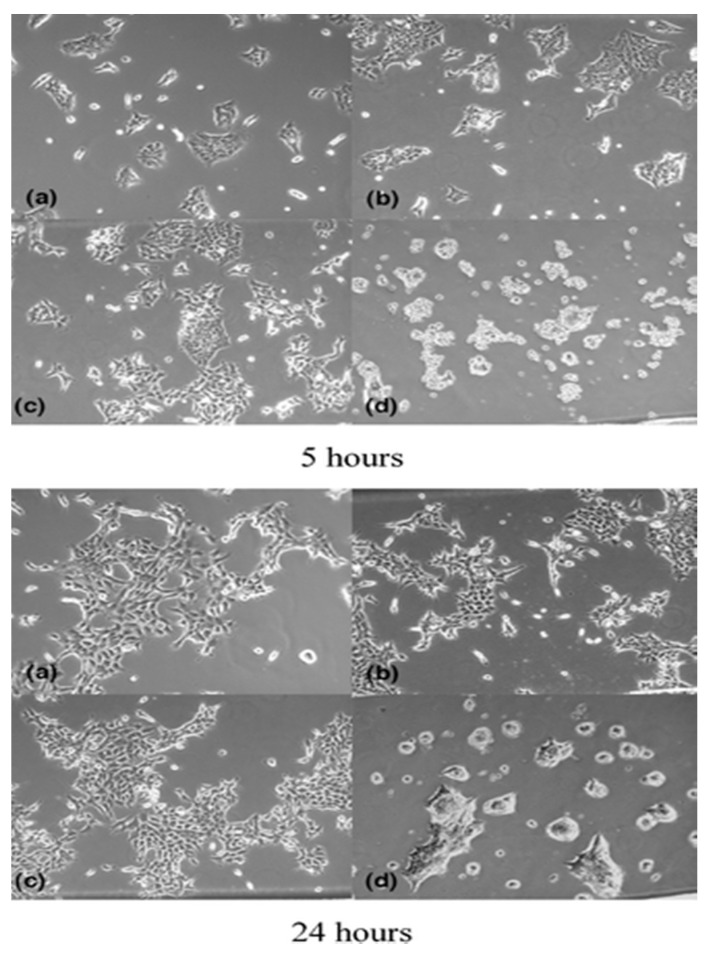
Phase-contrast images of NMuMG cells in various forms of cell culture. Panels (**a**–**c**) represent the NMuMG cells in standard forms of cell culture. Panel (**d**) represents the cells that were seeded in the developed gelation microfluidic device. Top and bottom subfigure sets are collected after 5 and 24 hours, respectively. Reprinted from [40] with permission. © 2006 Royal Society of Chemistry.

Phase-contrast imaging has also been used to image OOC platforms. Paguirigan et al. developed an enzymatically crosslinked gelatin microdevice for the use of cell culture [40]. The enzyme-crosslinked gelatin more closely mimicked the extracellular matrix than other in vitro methods, providing a more suitable microenvironment for cell growth [40]. Normal murine mammary epithelial cell line (NMuMG) cells were seeded into the device, and then quantitative phase-contrast images were taken to ensure the proper growth and morphology of the seeded cells [40]. It was found that the cells seeded in the gelatin microchannels developed a 3D structure, seen in Figure 3d, compared to the other control methods of cell culture where only monolayers of the cells were able to grow, as seen in Figure 3a–c [40]. As mentioned earlier, this leads to an advantage of this method: phase-contrast imaging allows for the morphological and phase changes to be analyzed in greater detail than with other imaging methods. This method also demonstrates the ability of phase-contrast imaging for other OOC platforms since the device discussed above was intended to closely mimic the in vivo growth of NMuMG cells and capture the 3D level details of the cells in the gelatin microchannel. One of the current directions of the OOC platform is in vitro drug tests that closely model an in vivo organ [41]. The developed OOC needs to model the disease of interest to test drugs effectively [41]. Huh et al. developed a lung on a chip platform to model pulmonary edema and imaged their platform through phase-contrast imaging [41]. These phase-contrast images exposed a space that was previously filled with air and later filled with a clear liquid as it leaked across the endothelial lining of the microvascular channel [41]. This depicts how phase-contrast imaging can be used to image OOC platforms towards one of the current trends of OOC applications.

### 2.5. Holographic Optofluidic Microscopy with Microfluidic Devices

**Figure 4:**
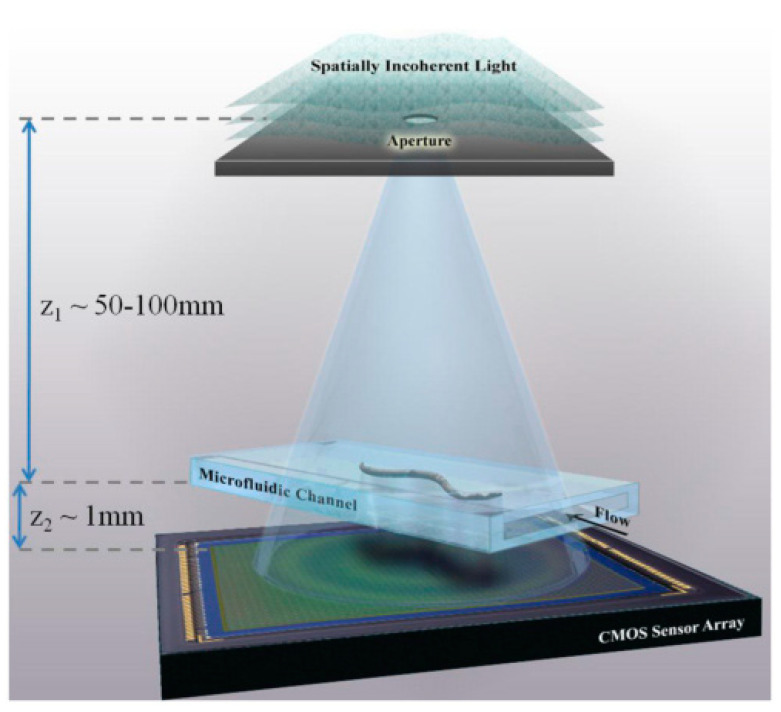
Experimental setup of conducting holographic optofluidic microscopy images of microfluidic devices. Reproduced from [43] under Creative Commons Attribution 4.0 License.

Holographic optofluidic microscopy is another form of transillumination imaging that can be used in microfluidic devices. It requires a quasi-monochromatic incoherent light source illuminated onto the microfluidic device, causing scattering and diffraction of light [42]. Such scattering and diffraction are used to produce the hologram at the sensor of the imaging set up, which can be seen in Figure 4 [43]. This imaging method is beneficial because both amplitude and phase images are obtained. Bishara et al. demonstrated how to produce high-resolution images of *Caenorhabditis elegans* using this imaging technique. Around 15 consecutive frames were sufficient for creating high-resolution images of the *C. elegans*. A computation algorithm is required to reconstruct these frames into an image. Some disadvantages include the signal-to-noise ratio that limits the pixel size of the sample that can be imaged at an acceptable resolution. Bianco et al. combined a microfluidic device with holographic imaging through two different grating designs on the microfluidic device, both parallel and orthogonal to the flow of the microfluidic channel [44]. The orthogonal design could extend the field of view, and the addition of the grating onto the microfluidic device platform allowed for a simpler, more portable device. The portability of this device was the main advantage, allowing point-of-care diagnostics. However, this holographic imaging method can be more complicated and does not offer as high of a resolution as other methods.

### 2.6. Holographic Optofluidic Microscopy with OOC Platforms

After extensive research, holographic optofluidic microscopy has not in the past or present been demonstrated to image an OOC platform. However, Bishara et al. demonstrated how holographic optofluidic microscopy could be applied to smaller micro-sized objects [43]. This not only lends to this imaging method’s application for microfluidic devices but also lays the foundation for OOC platforms. However, due to the downfalls of this method for being complicated and not producing high-resolution images, holographic optofluidic microscopy has not yet been demonstrated to image OOC platforms. High resolution imaging is needed to capture images of cells and subcellular structures with enough detail to illustrate what is occurring in the OOC platform. If the resolution of holographic optofluidic microscopy imaging can be improved, this method can potentially be applied to OOC platforms in the future.

## 3. Fluorescence Imaging

**Figure 5:**
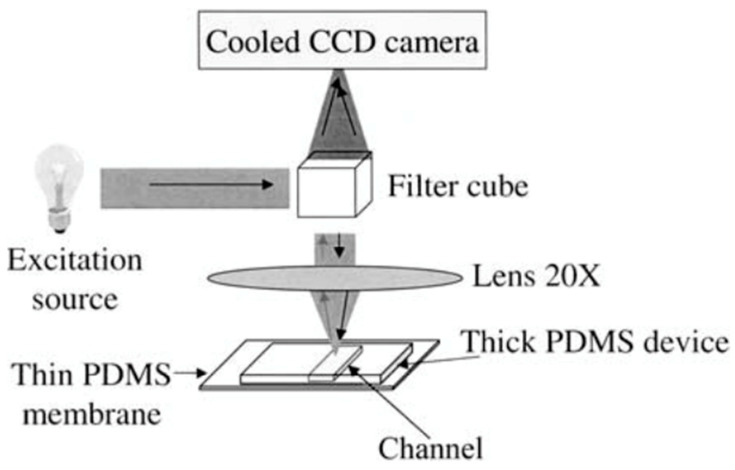
Schematic of the basic setup for fluorescence imaging where an epifluorescence microscope lens and CCD detection device are used. Reproduced from [44] with permission, © 2004 Springer Nature.

Fluorescence imaging, unlike transillumination techniques, requires the use of fluorophores to be conjugated to molecules to obtain an image [45,46,47,48,49]. The setup for this imaging method is similar to that of transillumination in that a microscope lens and a detector are needed, which in this imaging method are an epifluorescence microscope and a CCD detector as shown in Figure 5 [48]. The light travels through the device, bounces back through an objective lens, and hits a detector to form an image [48]. The main differences in fluorescent imaging comes from a different microscope lens being used, optical filters, and fluorescent labeling of the molecules. Therefore, it is label-based imaging method. The advantage of conjugating fluorophores to biomolecules is the ability to investigate a cellular and subcellular level of detail in the sample being imaged [23]. Such detailed observation is critical in monitoring mammalian cells’ successful proliferation and metabolism within OOC platforms [50,51,52]. In some cases, the fluorescence microscope can even be integrated onto the chip to allow for automatic imaging of the sample [4]. However, fluorescent probes are not available for all target molecules, meaning that some target molecules may not be imaged through fluorescence [53,54,55]. Fluorescent probes consist of a fluorophore attaching to a molecule and the selection of these probes require a lot of consideration to minimize complications such as photobleaching effects [56]. This means that there is not always a suitable fluorescent probe for the molecule of interest that will minimize these adverse effects [56]. In addition, fluorescent probes can also cause harm to the cell and prevent it from functioning properly [53,57,58,59]. Methods of fluorescence imaging used with microfluidic devices, like OOC platforms, include (1) confocal microscopies combined with fluorescence and (2) light-sheet fluorescence microscopy (LSFM).

### 3.1. Confocal Fluorescence Microscopy with Microfluidic Devices

Confocal microscopes combined with fluorescence imaging are used often in combination with microfluidic devices [60,61,62,63,64,65]. Confocal microscopy allows the acquisition of multiple 2-D images at different depths towards reconstructing 3D structures. The sample is optically sectioned, and the slice images of the sample can be obtained [23]. Addition of fluorescence imaging to the confocal microscopy provides the structural details at high resolution, which is beneficial for the imaging microfluidic platforms [60]. The downfalls to this method include photobleaching and phototoxicity of the sample [66]. Photobleaching reduces the time that sample is viable for imaging [67,68]. If this time is surpassed, the images will not reflect the actual state of the sample. Some studies have found methods that reduce the amount of photobleaching; however, photobleaching to any extent still presents an obstacle to obtaining high-quality images [69,70]. Overall, this imaging method offers good image resolution but suffers from photobleaching and phototoxicity to the samples.

### 3.2. Confocal Fluorescence Microscopy with OOC Platforms

**Figure 6:**
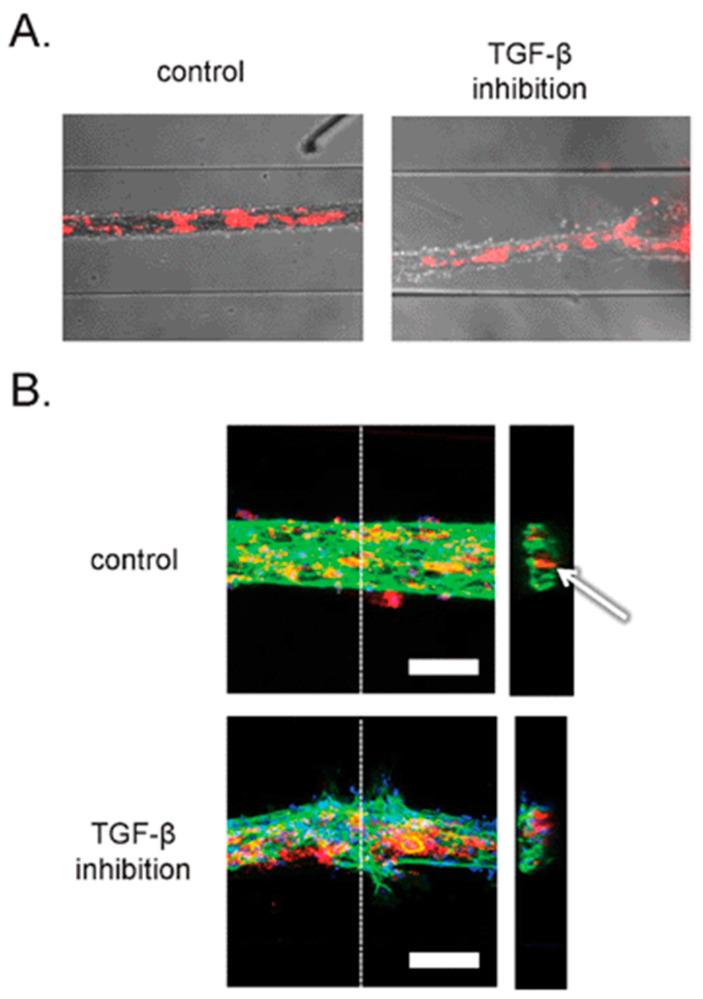
(**A**) Brightfield images combined with epifluorescence images of an OOC platform. (**B**) Confocal microscopy images taken from the same OOC platform to obtain a higher level of detail of the 3D structures and interactions within this OOC device. Reproduced from [32] with permission, © 2013 Royal Society of Chemistry.

When confocal microscopy is used with fluorescence imaging, cellular and subcellular details can be imaged over different depths of microfluidic channels, which is beneficial for OOC platforms [71]. This imaging technique can image various sized samples from single molecules to millimeter-sized samples [23]. Such single-molecule imaging is essential for OOCs, since the target molecules (subcellular details) are micron-sized. In this method, the microscopes can either be integrated onto the chip or as an external component to capture the fluorescence images [23]. Some examples of this imaging method were demonstrated in an OOC platform discussed earlier in this review. As discussed earlier, van der Meer et al. began with taking brightfield images of their OOC device to find that this form of imaging was incapable of imaging the cell-to-cell interactions [32]. In order to overcome this, confocal microscopy images of the fluorescently labeled cells were taken and found to produce a higher resolution than the brightfield images, as seen in Figure 6B [32]. Peel et al. followed a similar path in using confocal fluorescence microscopy after using brightfield imaging. As discussed earlier, Peel et al. used brightfield imaging on their liver on a chip device to define a field of view for their next step of higher resolution imaging which was confocal fluorescence microscopy [33]. Their imaging method was automated and could obtain single-cell resolution on their liver on a chip device [33]. This lends high resolution to be the significant advantage of confocal microscopy when used for OOC devices. The blood–brain barrier on a chip is also currently of importance to develop further due to its ability to study complex neurological disorders such as Alzheimer’s disease [72]. Herland et al. developed a blood–brain barrier on a chip, consisting of human brain microvascular endothelial cells, pericytes, and astrocytes [72]. Fluorescent images were taken to determine the correct 3D vessel structure formation in the microfluidic channels [72]. These images can depict the cell distributions within the blood–brain barrier on a chip lending to the high-resolution advantage of this imaging method [72]. Another current direction with OOC platforms is disease modeling such as cancer to test novel drugs and therapeutics. A specific tumor on a chip can be created to test therapeutics and drugs for the treatment of that tumor [73,74,75]. Fluorescent images can be taken from these tumor on a chip devices to ensure the formations of the correct 3D microvascular environment [73] and desired cell structure [74] before testing therapeutics and drugs in the device. Fluorescent images of a tumor on a chip device can also be taken to monitor how the cells mimicking the tumor can respond to a novel drug [75]. All of this depicts how fluorescence imaging can provide high-resolution images with cellular-level details from the OOC platforms, and how therapeutics and drugs affect these organ-like structures. This imaging technique holds the same disadvantages of photobleaching, phototoxicity, and a non-label-free method, as seen with other microfluidic devices.

### 3.3. Light-Sheet Fluorescence Microscopy (LSFM) with Microfluidic Devices

Light-sheet fluorescence microscopy (LSFM) combines the optical sectioning confocal microscopy with a high-speed laser scanning confocal over a large field of view [76,77,78,79,80,81,82]. Due of this combination, LSFM has advantages in a high acquisition rate with a reduced level of phototoxicity [83,84,85,86,87,88]. This high acquisition rate makes this method ideal for imaging OOCs allowing minimum phototoxicity to cells and high throughput screening [89,90]. Memeo et al. combined a microscope on a chip to image *Drosophila* embryos using LSFM [79]. They found that this imaging technique obtained a lateral resolution of 0.99 µm and 1.05 µm depending on the illumination wavelengths. Overall, LSFM benefits from a higher resolution than the other fluorescence imaging and a lower phototoxicity level, leading to high-quality images.

### 3.4. Light-Sheet Fluorescence Microscopy (LSFM) with OOC Platforms

**Figure 7:**
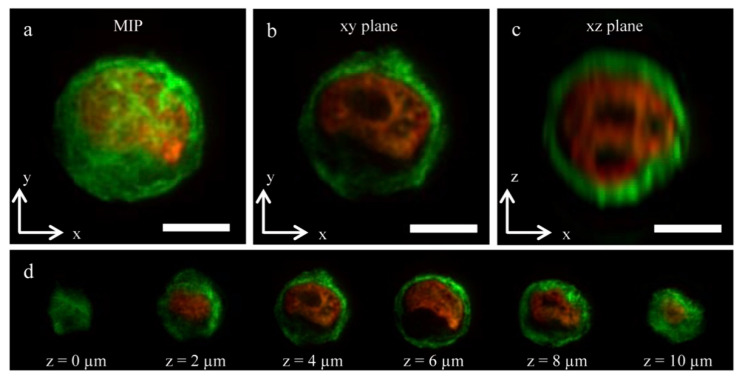
3D images captured in a microfluidic channel of human mammary epithelial cells taken in each 2D plane, panels (**a**–**c**). Panel (**d**) represent images of the mammary epithelial cells in an XY plane with various sizes of z being used to image. Reproduced from [91] under Creative Commons Attribution 4.0 License.

To date there have not been a substantial amount of LSFM being used to image OOC platforms. However, Sala et al. demonstrated the integration of a microscope onto their OOC device that could image through LSFM [91]. Their OOC platform was seeded with human mammary epithelial cells which are around 15 μm in size and the attached microscope was able to obtain high resolution images at a high acquisition rate of around 1 sample/s. They went on to further discuss that finer sampling of the samples could be obtained through reducing this acquisition rate. The importance of this work comes from the ability to obtain 3D images of the human mammary epithelial cells in their microfluidic device, as seen in Figure 7 [91]. This demonstrates this imaging method’s ability to obtain high-resolution 3D cell images, which is necessary for imaging OOC platforms. 3D cell images within a micro-size scale can provide important information on how the cells within an OOC are functioning and growing.

**Figure 8:**
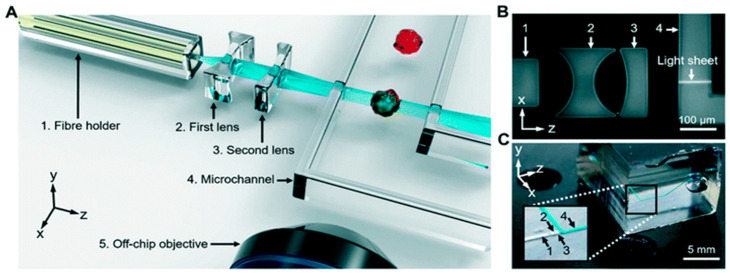
Panel (**A**) is a schematic of light-sheet fluorescence microscopy (LSFM) on a microfluidic device. Panel (**B**) represents a micrograph of the optical setup and panel (**C**) represents a photograph of the microfluidic device being imaged. Reproduced from [81] with permission, © 2021 Royal Society of Chemistry.

Another group, Vargas-Ordaz et al. focuses on single-cell 3D imaging in a microfluidic device [81]. They combined a submicron light-sheet within their microfluidic device. This design formed the light-sheet by focusing a laser beam into the microfluidic channel, as shown in Figure 8. A lateral resolution of 0.65 µm could be obtained, and that this method could be potentially beneficial due to this method’s low phototoxicity levels. Even though their device did not attach the cells in the microfluidic channels like commonly seen in OOC platforms, this work demonstrates the possibility to image cells at the micron scale using the LSFM technique. As seen in microfluidic devices, OOC platforms could benefit from this imaging method as it produces high quality images while reducing phototoxic effects commonly seen in fluorescent imaging. Not all phototoxic effects can be fully eliminated, however, leading to the drawback of LSFM used to image OOC platforms.

## 4. Smartphone-Based Imaging

**Figure 9:**
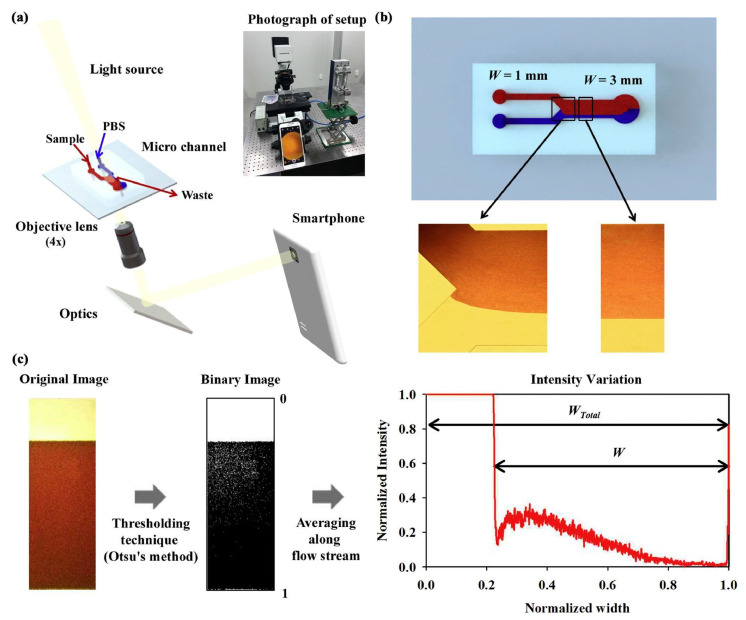
A smartphone microscope attachment (**a**) images a microfluidic device (**b**), testing viscosity of different samples using the image processing method (**c**). Reproduced from [94] with permission. © 2018 Elsevier.

Another form of imaging microfluidic devices and OOCs is the smartphone-based platforms. Smartphone-based imaging has been on the rise in recent years due to improvements in these devices’ hardware and software. These improvements have allowed better imaging of the samples [92,93,94,95,96,97]. A basic setup of smartphone imaging is provided in Figure 9a, where a light source, objective lens, smartphone, and microfluidic device are needed [94]. The light source travels through the microfluidic device, then travels through an objective lens to magnify the detail, and then hits the detector located in the smartphone to form an image [92]. In some cases, such as the lens-free-based smartphone imaging, the objective lens is not needed, which will be discussed further in Section 4.5 and Section 4.6. Advantages include the low cost, compared to traditional and portable imaging systems [93,98,99,100,101,102]. In addition, smartphone platforms can provide a point of care testing [93,103,104,105,106,107,108]. Such low cost and point of care testing are beneficial to provide testing in rural or underdeveloped areas, without expensive and sometimes bulky testing equipment. However, disadvantages lie within the smartphone’s default imaging application (app). Such apps are not designed for microscopic imaging. They perform numerous touch-ups to the images, such as spatial light bias adjustments, white balancing, localized focusing and defocusing, etc., which are inappropriate for imaging proteins and cells within microfluidic devices and OOCs. Therefore, apps must be further developed to obtain the best resolution images using smartphones [93]. Future software development and smartphone updates can provide this ability to process the images. Some imaging methods commonly combined with OOC platforms include microscope attachment-based smartphone microscopy, quantitative phase smartphone microscopy, and lens-free smartphone microscopy.

### 4.1. Microscope Attachment-Based Smartphone Microscopy with Microfluidic Devices

Smartphones can be combined with a microscope attachment that contains lenses and optical components, to serve as the microscope. The smartphone itself is used as the camera component needed to capture the images [93,94,109,110,111,112]. Kim et al. combined microfluidics, a smartphone, and a microscope attachment to measure the viscosity of various samples in a microfluidic device, as shown in Figure 9 [94]. Combining these three components allowed them to obtain highly accurate viscosity measurements through a small, portable device. The use of a microscope attachment and a smartphone on microfluidic devices enables low-cost microscopic imaging. Compared to commercial and standard imaging equipment, such as microscopes, the microscope attachment-based smartphone microscopy is a more cost-effective option. A disadvantage of this imaging method is the compromised image resolution compared to those from commercial benchtop microscopes. Navruz et al. developed a microscope attachment for a smartphone that contained LED transmission with a fiber-optic array [113]. They could obtain a resolution of around 1.6 µm with a field-of-view of >1.5 mm^2^. Submicron resolution could potentially be obtained with a denser fiber-optic array.

### 4.2. Microscope Attachment-Based Smartphone Microscopy with OOC Platforms

**Figure 10:**
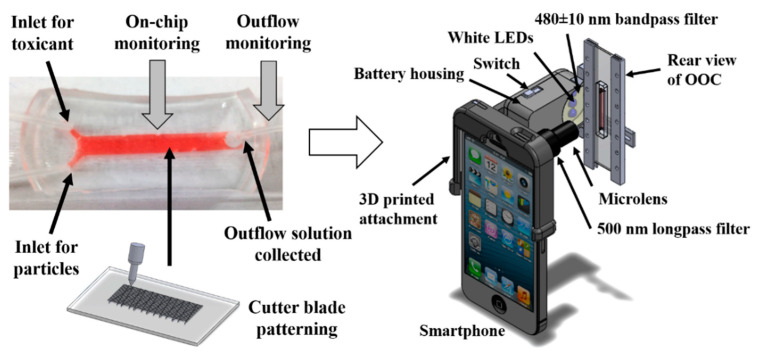
Schematic of how microscope-attachment based smartphone microscopy can be used to monitor both membrane expression and protein product release in a kidney on a chip device. Reprinted from [4] with permission. © 2016 Elsevier.

There has been some recent research on the use of a microscope attachment-based smartphone microscopy with OOC platforms. Yafia et al. used a smartphone with an attachment that contains a miniaturized microscope lens [111]. This work was in its preliminary stages and had not yet been extensively tested with microfluidic devices. However, some experiments were completed to image droplets where conclusions were made that future work could be conducted to integrate onto an OOC device. They also confirmed that this imaging method is a low-cost option compared to traditional benchtop microscopes. Another group, Cho et al., developed a smartphone-based fluorescence microscope to monitor their OOC device [4]. The OOC platform was a kidney on a chip device to observe how nephrotoxic drugs reacted with the device, one of the current trends seen within the OOC applications, which would give an approximation on how these drugs would respond in a human kidney [4]. This smartphone-based fluorescence microscope was attached directly to the kidney on a chip device to allow for in situ monitoring and dual-mode detection, which is beneficial to allow for real-time monitoring [4]. The dual-mode detection is explained in Figure 10, where both nanoparticle immunoagglutination and particle capture can be monitored with this imaging method [4]. Furthermore, they could observe both membrane expression and protein product release in this OOC platform with microscope-attachment-based smartphone microscopy [4]. While this dual detection imaging has not been demonstrated in other OOC platforms, this example shows the future potential of this imaging method to monitor a good level of detail in what is occurring within the OOC platforms. Overall, these smartphone-based microscope attachments benefit from low cost but suffer from lower resolution compared to benchtop microscopes.

### 4.3. Quantitative Phase Smartphone Microscopy with Microfluidic Devices

Quantitative phase microscopy in conjunction with smartphones provides images of quantitative cellular phases. This method can create high-contrast and high-quality images [114]. In addition, quantitative phase imaging offers label-free imaging, leading to less destruction of the sample, such as seen in fluorescence imaging [115]. Meng et al. used this method by combining a light source, micro-objective, and an eyepiece with a smartphone to develop their quantitative phase microscope [114]. They combined their developed platform with an app and computational algorithms to compute the phase distributions. This imaging platform could image various samples and produce high-resolution images. Yang and Zhan used quantitative phase imaging to image blood cells and obtain high-resolution images of around 1 µm [115]. This method reduces the overall cost compared to the quantitative phase microscopy using transillumination since objective lenses are not needed, and the lens of the smartphone can be used.

### 4.4. Quantitative Phase Smartphone Microscopy with OOC Platforms

**Figure 11:**
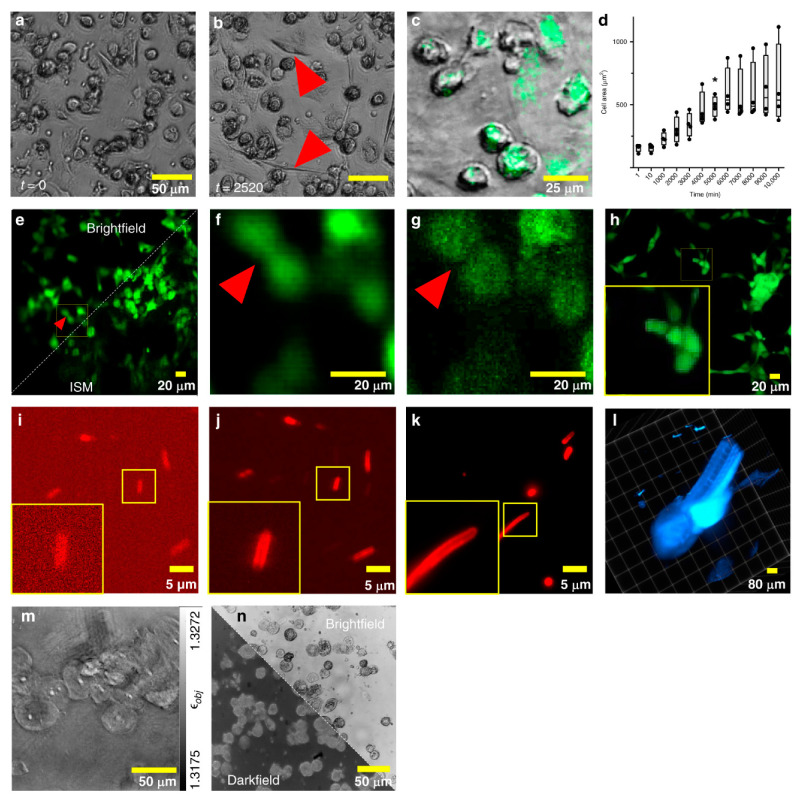
Diedrich et al. demonstrated the ability to continuously monitor morphological changes in macrophages with a smartphone camera, shown in panels (**a**–**c**). Panel (**d**) represents the growth of the differentiating cells at various time points where panels (**e**–**n**) represent various other types of images taken of the cells throughout this study. Reproduced from [116] under Creative Commons Attribution 4.0 License.

Quantitative phase microscopy can also be used with OOC platforms. Diederich et al. demonstrated imaging of morphological changes in macrophages in an OOC device, as shown in Figure 11 [116]. In order to obtain these images, a setup was developed that featured a smartphone and some 3D-printed attachments [116]. Differences in the morphology of a macrophage can be important indicators to the possible detection of pathogens or the potential for phagocytosis to occur [116]. This can be important for OOC platforms as the ability to monitor various cellular level morphology changes can predict the current status of those cells. They demonstrated the capability of this imaging method to capture the morphology level detail. However, it was found that a focus drift occurred when acquiring these images due to temperature-dependent deformation [116]. Nonetheless, the morphological changes could be imaged, which can be beneficial when the changes in cell structure are of importance to the study. In addition, as Section 4.3 states, this imaging method also benefits from lower costs than quantitative phase microscopy using transillumination.

### 4.5. Lens-Free Smartphone Microscopy with Microfluidic Devices

**Figure 12:**
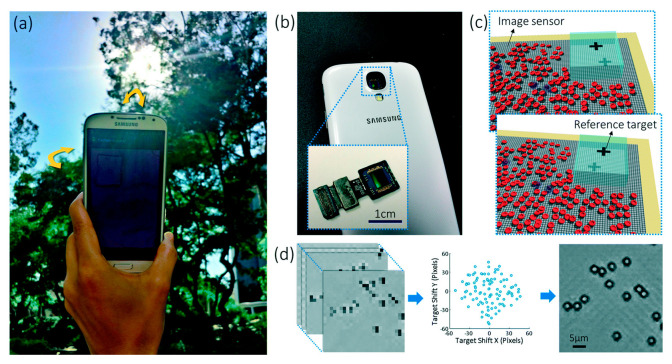
Lens-free microscopy example on a smartphone. The ambient light was used as the light source shown in panel (**a**) and the image sensor from the smartphone was used to capture images seen in panel (**b**). The samples were placed directly onto the imaging sensor of the smartphone shown in panel (**c**). Images of the cells are shown in panel (**d**). Reproduced from [118] with permission. © 2014 Royal Society of Chemistry.

Lens-free microscopy with a smartphone has been a more recent area of study, as it can potentially reduce the cost and size of imaging platforms. Traditional microscopes are large in size and high in price [117,118,119], which becomes an issue for point-of-care and field-based imaging [118,120]. Lens-free imaging requires no lens, lasers, or other optical components and can be attached directly to a smartphone to image [120]. A simple light source, such as a light-emitting diode (LED), is used to illuminate the samples and is passed through a large aperture [120]. This filtered light will interact with the sample in which the scattering of this can be detected through the CMOS array that already exists in the smartphone camera [120]. After detection, an image can be reconstructed of the sample [120]. Lee and Yang developed a lens-free imaging platform and did not require a dedicated light source [118]. In their platform, ambient light was used to illuminate the sample. The sample was placed directly onto the image sensor of a smartphone, and then ambient light was used to create shadow images, as shown in Figure 12. After a sequence of images is collected at varying angles of light, an app is then employed to reconstruct a high-resolution image of the sample. The downfall to this method is that higher resolution imaging results in slower processing speed and more extensive data size [118]. However, overall, this is a relatively low-cost and straightforward option for capturing micron to nanometer scale images. Guan et al. demonstrated another potential imaging method using a smartphone without a microscope attachment to capture images of a paper-based blood assay microfluidic device [121]. In this imaging technique, a smartphone captured the images of the blood typing assay, and then an app was developed to analyze the eluting length information found on the assays. After the image was processed in the app, the app would display the blood typing result on the smartphone screen. This imaging method allows for rapid analysis and diagnostic capabilities combined with a paper-based microfluidic device and a smartphone. In addition, this imaging technique could be beneficial to OOC platforms as this method demonstrates the ability of a smartphone to perform the steps of image capture and analysis, all without using a microscope attachment.

### 4.6. Lens-Free Smartphone Microscopy with OOC Platforms

**Figure 13:**
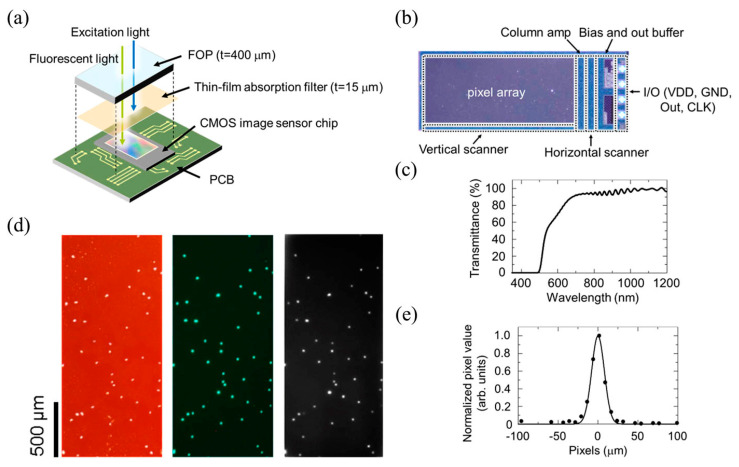
Breakdown of the lens-free on-chip CMOS fluorescent imager, panel (**a**). Panel (**b**) is an image of the CMOS-image sensor ship used. Panel (**c**) is the transmittance spectrum of the ultra-thin glass bottom microfluidic device. Panel (**d**) are brightfield and fluorescent images of the cells using a table-top microscope in the left and middle image and the CMOS fluorescent imager was used to capture the image of the cells on the right. Panel (**e**) are intensity profiles captured of the microsphere. Reproduced from [122] under Creative Commons Attribution 4.0 License.

**Figure 14:**
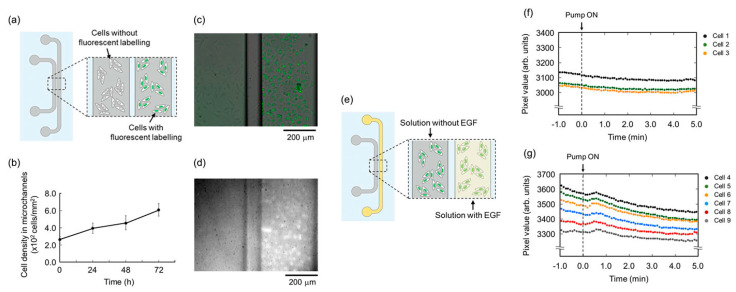
Panel (**a**) represents the cells in the microfluidic channels and panel (**b**) represents the number of cells in the microchannels as a function of time. Demonstration of lens-free on chip fluorescence imaging HeLa cells seeded on collagen treated microchannels, panels (**c**,**d**). Panel (**e**) is a schematic of EGF delivered to the microchannels, panel (**f**,**g**) are the time course to detected fluorescence of the cells being imaged. Reproduced from [122] under Creative Commons Attribution 4.0 License.

Lens-free microscopy with a smartphone has not been demonstrated extensively for imaging OOC platforms. This imaging technique requires the sample to be close or on top of the imaging sensor in the smartphone [118,120]. This decreases the feasibility of imaging OOC platforms as the cells are typically seeded into the microfluidic device [40]. In order for this to work, the cells would have to be able to be imaged through the microfluidic device which is typically constructed from PDMS [25]. To get around this, Takehara et al. implemented the use of an ultra-thin glass bottom microfluidic chip so that lens-free on-chip fluorescence imaging could be used [122]. HeLa cells were seeded onto collagen-treated microchannels and then imaged using a contact CMOS fluorescent imager, in which the setup can be seen in Figure 13 [122]. This imaging setup consisted mainly of a CMOS image sensor, thin-film absorption filter, and a fiber optic plate (FOP) to protect from damage and maintain a flat surface for the imaging setup [122]. Figure 14 shows the images of these cells that were obtained with this imaging setup [122]. Of importance to note, Figure 14f,g represent the activity of the cells without being treated with endothelial growth factor (EGF), Figure 14f, and the activity of the cells treated with EGF, Figure 14g, where an increase in fluorescent signal is observed in the cells treated with EGF [122]. This demonstrates the ability of this imaging method to monitor changes in cells in an OOC platform. This imaging method is beneficial as a low-cost option to monitor how seeded cells change in response to various biomolecules. However, this method does suffer from decreased resolution as the distance between the sample and the imaging sensor increases [122]. This resolution can be improved by reducing this distance. Overall, this imaging method is fairly new to imaging OOC platforms; however, there are advantages to using this method as stated earlier.

## 5. Conclusions

Table 1 summarizes the strengths and weaknesses of a variety of imaging methods discussed in this review. Transillumination methods, including brightfield, phase-contrast, and holographic optofluidic imaging, suffer from low resolution, which is not suitable for imaging the cells and subcellular structures within OOCs. Despite this setback, brightfield and phase-contrast imaging have been demonstrated for OOCs, presumably due to their simple operation principles. Phase-contrast imaging may deserve further investigation due to its ability to measure phase and morphological changes of mammalian cells, if its resolution can be increased substantially. Holographic optofluidic imaging also deserve further investigation due to its portability and phase imaging capability; however, its operation should be simplified and resolution should further be improved for OOC applications. Fluorescence imaging, including confocal fluorescence and LSFM, has suffered from photobleaching and phototoxicity to the mammalian cells. Despite these disadvantages, they have nonetheless been used for OOC platforms. LSFM deserves more investigation, as it has high acquisition rate leading to reduced photobleaching and phototoxicity. Smartphone-based imaging, including microscope attachment-based, quantitative phase microscopy, and lens-free microscopy, is the most promising method since they provide low-cost, excellent portability, and potentially high resolution (compared to transillumination imaging). Quantitative phase smartphone microscopy can measure phase and morphological changes, while it suffers from focus drift. Lens-free smartphone microscopy is potentially the most promising method due to its simplicity and low cost. However, it has not been demonstrated extensively at this point in time for OOCs, as the mammalian cells within OOCs must be placed very close to the camera. If the smartphone camera or other CMOS camera can be integrated into or very close to the OOCs, it can successfully be implemented and more often used with OOC platforms in the future.

As summarized in this review, various imaging methods can be used to image samples in microfluidic devices, as well as OOCs. There are some imaging methods discussed in this review that are not currently compatible with OOCs. Holographic optofluidic microscopy has not yet been demonstrated to image OOC platforms. This is due to the lower resolution that this imaging method provides. OOC platforms requires micron scale resolution, meaning that higher resolution is needed to capture images from OOC platforms. In addition, lens-free smartphone-based microscopy has not been demonstrated extensively for imaging OOC platforms. This is due to this imaging method requiring the sample to be placed onto or as close as possible to the imaging sensor in the smartphone. For this imaging method to be used more widely in OOCs in the future, the chip would have to be designed with a material that would allow the seeded cells to be imaged through that material from the imaging sensor on the smartphone such as an ultra-thin glass microfluidic device as discussed in Section 4.6. Overall, some methods are focused on higher resolution, others are focused more on low cost and ease of transport. As microfluidic devices and OOCs are low cost and can be transported easily, imaging on microfluidic devices, and OOCs should also be made low cost and portable. Furthermore, as technology advances, there could also be improvements in smartphone-based imaging methods, since these methods often rely on an app or computational algorithm to analyze the images. This means that there should be advancements in the resolution provided by smartphone-based imaging with OOC platforms in the future. Due to the low cost and transportability benefits, smartphone-based imaging could become a widely used imaging method for OOC platforms in the future over the other imaging methods discussed in this review. Finally, imaging methods that can be integrated onto the OOC device can be beneficial for real-time and in situ monitoring of the sample.

## Figures and Tables

**Table 1:** Summary of the imaging methods discussed in this review and their strengths and weaknesses when used to image microfluidic devices and OOC platforms.

Method	Sub-Method	Strengths for Microfluidic Devices	Weaknesses for Microfluidic Devices	Strengths for OOCs	Weaknesses for OOCs	Ref.
Transillumination	Brightfield	Ability to automatically count cells	Blur in images; need for traditional microscope; less cost-effective; not transportable	Preliminary method before higher resolution imaging	Low resolution	[26,27,30,31,32,33]
	Phase-contrast	Extended field depth; Ability to measure phase changes	Low resolution	Ability to measure phase and morphological changes	Low resolution	[39,40]
	Holographic optofluidic	Phase images can be obtained; Allow portability to device	High signal-to-noise ratio; complicated; decent but not the best resolution	None at this time	Not currently demonstrated in OOC platforms	[42,43]
Fluorescence	Confocal	High resolution	Photobleaching; phototoxicity; not label-free	High resolution; ability to measure cell–cell interactions	Photobleaching; phototoxicity; not label-free	[32,33,45,46,47,48,49,66]
	LSFM	High acquisition rate; reduced phototoxicity	Some phototoxicity still exists	High acquisition rate; reduced phototoxicity	Some phototoxicity still exists	[23,81,83,84,85,86,87,88,91]
Smartphone-based	Microscope attachment-based	Low-cost; portability	Compromised resolution compared to benchtop microscopy	Low cost; in situ monitoring	Compromised resolution compared to benchtop microscopy	[4,111]
	Quantitative phase microscopy	Ability to measure phase and morphological changes; label-free; high resolution; reduced cost	Only suitable for imaging phase changes	Ability to measure phase and morphological changes; label-free	Focus drift can occur	[114,115]
	Lens-free microscopy	Reduced cost and size; high resolution	Slow processing speed	Low cost; can monitor cell activity in response to biomolecules	Lower resolution if image sensor is not close to cells	[118,120,122]
